# Response of Novel Functionally-Graded Prepacked Aggregate Fibrous Concrete against Low Velocity Repeated Projectile Impacts

**DOI:** 10.3390/ma14020280

**Published:** 2021-01-07

**Authors:** Nandhu Prasad, Gunasekaran Murali, Roman Fediuk, Nikolai Vatin, Maria Karelina

**Affiliations:** 1 School of Civil Engineering, SASTRA Deemed University, Thanjavur 613401, India; nandhuprasad@sastra.ac.in; 2 Polytechnic Institute, Far Eastern Federal University, 690922 Vladivostok, Russia; fedyuk.rs@dvfu.ru; 3 Higher School of Industrial, Civil and Road Construction, Peter the Great St. Petersburg Polytechnic University, 195251 Saint Petersburg, Russia; vatin@mail.ru; 4 Department of Machinery Parts and Theory of Mechanisms, Moscow Automobile and Road Construction University, 125319 Moscow, Russia; Karelinamu@mail.ru

**Keywords:** projectile impact, steel fibres, polypropylene fibre, functionally-graded concrete, preplaced aggregate

## Abstract

Preplaced Aggregate Fibrous Concrete (PAFC) is a newly minted composite that has recently become more popular. The production of PAFC involves two essential processes; first, the fibres and coarse aggregate were filled into the empty framework to form the first layer of a natural skeleton, followed by grout injecting. A cement grout fills the voids in the first layer skeleton with slight compaction. This process is repeated to complete the remaining layers; hence, a type of Functionally-graded Preplaced Aggregate Fibrous Concrete (FPAFC) is obtained. The most recent studies revealed that the literature regarding the high-velocity projectile impact on fibrous concrete is well documented; however, the low-velocity repeated projectile impact on PAFC is still unexplored and needs particular emphasis. This research aims to investigate the FPAFC made with a new type of steel and polypropylene fibres against low-velocity projectile impact to fill this research gap. In the current study, twelve mixes were prepared with mono and hybrid combinations of fibres for pioneering the possible utilization of fibres in FPAFC. The maximum fibre dosage in this study is limited to 2.4%. The projectile impact resistance of FPAFC was assessed in line with penetration depth, front and rear damage surface area, weight loss, damage ratio and failure pattern. Additionally, a simplified analytical model was introduced to compute the ejected composite mass from the tested specimens. The results revealed that the addition of steel fibre in a single layer FPAFC exhibited an increasing compressive strength trend compared to the two/three-layered FPAFC. Furthermore, increasing the dosage of fibre at the bottom and top layers of FPAFC with a hybrid combination alleviates the spalling with an increasing number of impacts. The results from this research offer the reference information for more detailed research and studies of FPAFC under low-velocity projectile impact.

## 1. Introduction

Precision guided weaponry has the capacity of numerous strikes, resulting in protective structures being subjected to multiple impacts. Protection of these structures under impact loading has become a significant, decades-long matter of public interest. However, fibre reinforced concrete is used more often in civilian and defence infrastructure against extreme impact loading; they still did not have inadequate strength under low velocity repeated impacts. Thus, with the new technological development and materials, there is a rise in the demand for new building material evolution with exceptional impact resistance.

PAFC is a newly minted concrete; it also called injected aggregate concrete, grouted aggregate concrete, prepacked concrete, Colcrete, Polcrete, Naturbeton, Arbeton and two-stage concrete [1]. The production technique of PAFC is different from conventional fibre reinforced concrete. First, the fibres and coarse aggregate were packed into the formwork to form the first layer of a natural skeleton, followed by grout injecting [2,3,4], which allows more coarse aggregates and fibres to be packed into the empty formworks and to interlock each other [5]. Increasing the content of coarse aggregate on PAFC changes the concrete properties. For example, these coarse aggregates’ contact points were increased due to their interlocking [6], resulting in better stress distribution during loading [7,8]. However, the coarse aggregate segregation can be eradicated using this unique PAFC casting procedure. This technology allows the achieving of dense monolithic concrete without any vibration of compaction, resulting in energy saving and labor costs [1]. PAFC has been extensively used in several applications such as high-density coarse aggregates to shield radiation, underwater structures, tunnels and mass concrete structures [9]. This wide range of usability is due to its sustainable advantages and cost-benefits.

Plenty of investigations have been performed to investigate the impact response of PAFC against drop weight impact. However, only limited studies are available on the projectile impact response of PAFC. Abirami et al. [10] examined the projectile impact response of three-layered PAFC comprising steel and glass fibre mesh. Twenty composite mixes were prepared, and all specimens were reinforced with steel fibre at 2.5% dosage with glass fibre mesh inserted between the concrete layers. The effect of various diameters (50, 75, 100, 125 and 150 mm) and the number of glass fibre mesh insertions (1 to 4) between the layers were studied extensively. Findings revealed that the 150 mm diameter of glass fibre mesh insertion in PAFC increased the number of impacts and significantly alleviated the penetration depth, and top and bottom surface damage area under projectiles. Murali and Ramkumar et al. [11] examined the impact performance of low carbon cementitious layered PAFC derived from calcined clay, fly ash, clinker and steel fibres. The finding revealed that the layered PAFC exhibited considerable improvement in the number of blows causing initial cracking and ultimate cracking by about 561% and 1804%, respectively, compared to plain concrete. A superior impact performance was noted in layered PAFC compared to one-layer PAFC with the same fibre dosage amount. Ramprasad and Murali [12] investigated the three-layered PAFC slab comprising a hooked end and crimped steel fibre at each layer’s different dosage and compared it with a one-layer PAFC slab. The three-layer PAFC slabs were reinforced with a dosage of 4, 2, and 4% steel fibre for the bottom, middle, and top layers. The findings revealed that the layered PAFC slab delivered exceptional impact resistance compared to the one-layer PAFC slab containing the same amount (3.33%) of steel fibre.

A new type of non-homogeneous concrete is called functionally graded concrete, which has varying structural properties from one side to the other. Because of this, a higher fibre dosage is used only in the top and bottom layers of structural concrete against impact loading under compression and bending [12,13]. Quek et al. [14] studied the small projectile impact response of functionally graded cementations panels (FGCP) under high-velocity. Results revealed that the excellent resistance to impact was noted in FGCP compared with a panel made with plain mortar. Consequently, plain mortar panels were broken into many pieces, and FGCP was continued unbroken when the projectile’s velocity was above 300 m/s. Mastali et al. [15] studied the functionally graded reinforced concrete by changing each layer’s fibre dosage. Findings revealed that the penetration depth, and the damaged surface area at the bottom and top, were significantly reduced compared to the traditional fibrous concrete comprising an equal amount of fibres. Moghadam et al. [16] reported that functionally graded reinforced concrete slab with nonuniform fibre distribution could alleviate the projectile penetration depth. Moghadam et al. [17] investigated the slab prepared with functionally graded concrete subjected to falling mass and projectile impacts. Sixty slabs of 400 × 400 × 75 mm were cast, which were reinforced with a 1% dosage of nylon and steel fibres. Slabs were prepared according to the concept of fully reinforced with uniform fibre distribution and functionally graded reinforced concrete with nonuniform fibre distribution. The findings indicated that the steel fibres exhibited an excellent projectile impact resistance compared to nylon fibre. In addition, functionally graded reinforced concrete slabs displayed an exceptional performance in reducing the depth of penetration and destroyed volume compared to fully reinforced concrete slabs.

In this study, the FPAFC made with a new type of polypropylene and steel fibres against low-velocity projectile impact is investigated. Twelve mixes were prepared with mono and hybrid amalgamation of fibres for pioneering the potential utilization of fibres in FPAFC. The maximum fibre dosage in this study is limited to 2.4% in all designed mixes. The study parameters such as penetration depth, bottom and top damaged surface area, ratio of damage and failure pattern were examined.

## 2. Experimental Program

### 2.1. Used Raw Materials

**Figure 1:**
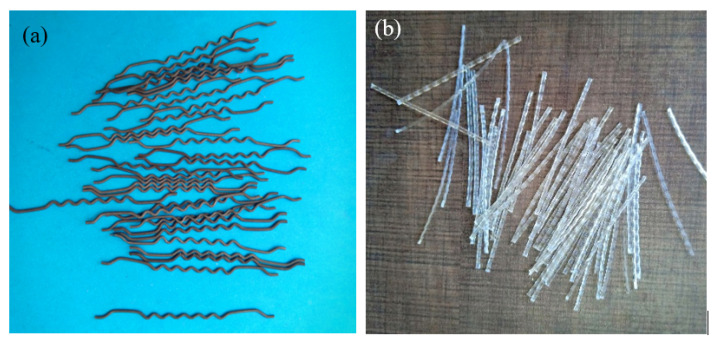
Type of fibre: (**a**) steel and (**b**) polypropylene.

- The Portland Pozzolana Cement was utilized as per IS:1489-1991 [18] and obtained from Dalmia cement.
- The natural river sand was utilized as fine aggregate with the values of 2.41 fineness modulus and 2.65 specific gravity, in accordance with IS: 383-2016 [19].
- A 12.5 mm size of coarse aggregate was utilized as per IS: 383-2016 [20]. An apparent bulk density, specific gravity and water absorption of coarse aggregate were 1700 kg/m^3^, 2.69 and 0.56%, respectively.
- A commercially available water reducing agent (superplasticizer-SP), namely Tech mix, 640 was utilized to obtain the flowable grout. The water reducing agent dosage is differed from 0.3% to 0.4% by weight of cement.
- A new type of fibre, namely macro polypropylene, and a hybrid crimped-hooked end were utilized. The polypropylene fibres (PF) and steel fibres (SF) appearance are exemplified in Figure 1. The length, diameter, aspect ratio and tensile strength of PF were 45 mm, 0.8 mm, 56 and 500 MPa, respectively. Likewise, the length, diameter, aspect ratio and tensile strength of SF were 50 mm, 1 mm, 50 and 1150 MPa, respectively. The density of SF and PF were 7850 and 910 kg/m^3^, respectively. The PF is sourced from the Kalyani Polymers Pvt Ltd., Bangalore, India and the SF from Purushothaman steels, Nagpur, India.

### 2.2. Mixing Proportions

**Figure 2:**
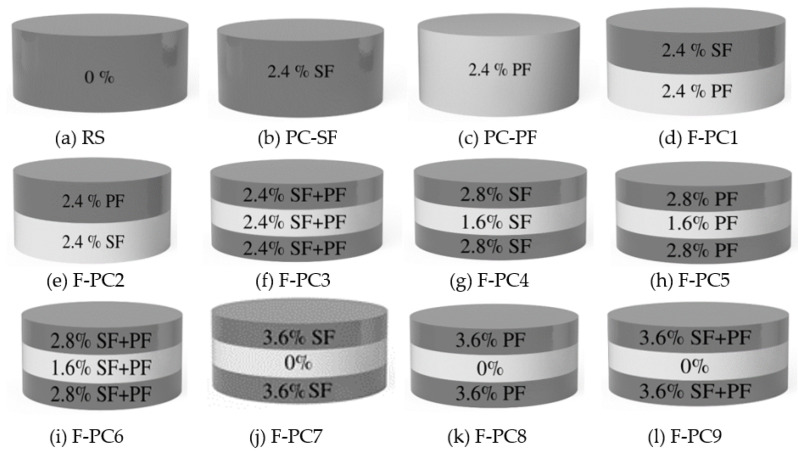
Dosage of fibre introduced in each layer of concrete. (**a**) RS (**b**) PC-SF (**c**) PC-PF (**d**) F-PC1 (**e**) F-PC2 (**f**) F-PC3 (**g**) F-PC4 (**h**) F-PC5 (**i**) F-PC6 (**j**) F-PC7 (**k**) F-PC8 (**l**) F-PC9.

In this study, twelve mixes were prepared, the ratio of cement to sand (c/s) was 1.0 and the ratio of water to cement (w/c) was 0.45 selected based on the efflux time test. These optimized ratios were used to prevent honeycombing and improve cement grout penetration between the fibre and aggregate skeleton voids. A commercialized high quality water reducing agent was used to flow grout freely, which leads to filling voids effectively in the aggregate skeleton through the method of gravity. Several trial testings were conducted to achieve the grout flows freely and meets an efflux time from 35 to 40 ± 2 s in line with ASTM C939/C939M—16a [21] and ACI 304.1 [22]. Twelve mixes were prepared with different amalgamations of PF, SF and hybrid combination at 2.4% dosage by volume of concrete. The first mixture was prepared with conventional plain concrete, considered as reference concrete (RC). The second and third mixes were denoted as PC-SF and PC-PF, respectively, and SF and PF were used with a dosage of 2.4%. Two-layer was introduced in the fourth and fifth mixes of FPAFC containing SF and PF at top and bottom in the fifth mix, while PF and SF at top and bottom in the fifth mix. The three-layered concrete scheme was introduced in the remaining mixes by changing the dosage and combination fibres at each layer. Table 1 demonstrates the mixed composition of FPAFC. Figure 2a–l illustrates the amount of fibre used in each layer for producing FPARC. The SF dosage in PAFC is usually limited to 6% because of the presence of coarse aggregates. Hence, SF with higher dosages (i.e., more than 6%) tend to cause a higher amount of fibres to be occupied in the mold, which turns into a concept of slurry infiltrated concrete. In this study, the PF density is significantly less (900 kg/m^3^) than the SF (7850 kg/m^3^). The 6% dosage of PF leads to more fibres not possible to accommodate into the mold. Hence, the fibre dosage used in this study is limited to 2.4%. The combination of fibre has been chosen based on an earlier study [13].

### 2.3. Procedure for Specimen Preparation

**Figure 3:**
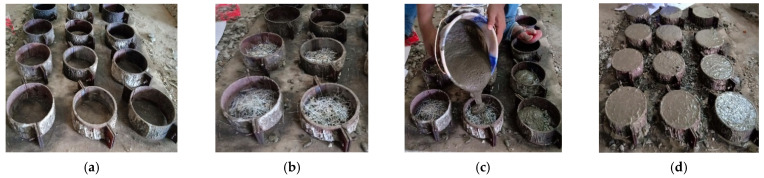
FPAFC casting process: (**a**) empty formwork, (**b**) packed fibres and coarse aggregates in formwork, (**c**) pouring of grout, (**d**) finished specimens.

Concrete discs of cylindrical shape which have a thickness of 64 mm with diameter 152 mm, were casted to assess the FPAFC resistance to the projectile impact. The cylindrical disc was separated into three layers. The middle layer designed thickness was 20 mm, while the 20 mm thickness was maintained at bottom and top layers. Initially, a steel formwork/mold was placed on a flat surface and it was sealed completely to be free from leakage, which enabled the cement grout to penetrate freely to fill voids in the skeleton. Then, the cement grout was slowly poured on the first layer. The penetration of grout was good in this process and able to fills the voids completely between the coarse aggregates and fibres. In this method, a layer is subjected to slight compaction to prevent honeycombing. Subsequently, a similar process was adopted to complete the remaining two layers. All casted specimens were subjected to room temperature curing. After 24 h, the demolding process was initiated for immersion curing for 28 days prior to testing. Figure 3 illustrates the FPAFC process of casting. Furthermore, the 28 days compressive strength of FPAFC obtained from 100 mm cubes according to IS 516 [23].

### 2.4. Projectile Impact Test

**Figure 4:**
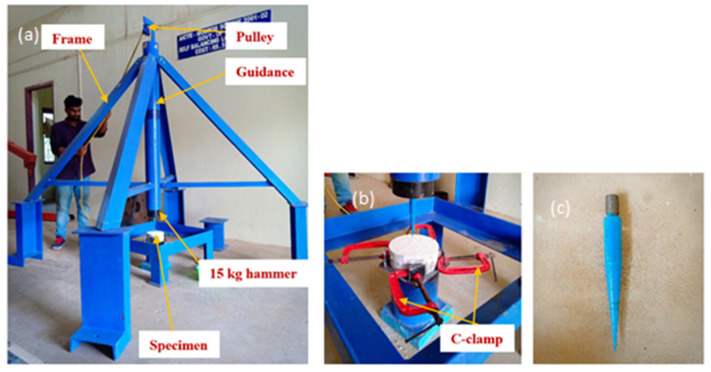
Projectile impact testing device. (**a**) Testing device (**b**) specimen arrangement (**c**) projectile needle.

Figure 4 illustrates the low-velocity projectile impact testing setup, C-clamp compound bevel needle. The projectile impact needle was prepared using EN8 engineering steel (non-deformable steel). The compound bevel needle is fixed into the hammer of 15 kg and the height and diameter of the compound bevel were 150 and 20 mm, respectively. The hammer was repeatedly dropped from a height of 0.5 m on to the specimen centre until the needle pierced and appeared on the specimen’s rear surface. The FPAFC specimens were placed on a steel stool with four standing lugs to ensure that the projectile was striking in the same spot repetitively. Four positioning steel lugs and C-clamps were used to inhibit the specimen movement. The needle was striking the FPAFC specimens with a velocity of 5 m/s per blow. The hammer weight and drop height have been maintained based on our earlier study [24]. The depth of penetration, weight loss, and the damaged area at the front and rear surfaces were noted for every four blows. The digital vernier caliper was used to measure the damaged surface area’s diameter at the front and rear surfaces. The penetration depth was measured with a slim needle inserted into the punctured hole.

## 3. Discussions of Experimental Test Results

### 3.1. Compressive Strength of FPAFC

Table 2 demonstrates the observed compressive strength of FPAFC specimens. The direction of the loading in a compressive test is applied across the layers. The applied load was at the top surface of the specimen’s top layers, and a failure load was noticed. It can be observed prudently from Table 2 that there is an increasing trend of compressive strength in all fibrous specimens compared to RC. The addition of mono SF with single-layered PC-SF specimens exhibited an intrinsic increase in compressive strength by about 59.5% compared to RC. In contrast, the PC-PF specimen exhibits only 18.7% improvement in compressive strength. The best contribution comes from SF, while the PF had significantly less effect in strength enhancement. This phenomenon is due to the high tensile strength of SF and fibre bridging action, which hinders the cracks’ extension, leading to a delay in the crack development rate [25].

On the other hand, the two-layered FPAFC concrete specimens (F-PC1 and F-PC2) exhibit 26.1 and 23.0% enhancement in compressive strength as associated with RC. A small change was noted in the strength between these two specimens due to the addition of SF at the top layer, which shows the improved behaviour. This is ascribed to stress concentration at this particular region being significantly alleviated, which leads to arresting cracks by interlinking micro and macro cracks resulting in higher strength [24]. It is evident from Table 2 that the FPAFC specimen with three layers exhibited an enhancement in compressive strength as associated with the RC specimen. Hybridization of fibre leads to great enhancement in compressive strength. For instance, the F-PC3 specimen incorporated by 2.4% of SF+PF combination had an increasing trend and the strength value increased by about 48% as compared to RC. The enhancement is ascribed to the SF+PF presence, which plays a crucial role in arresting cracks and their proliferation. The minimal compressive strength was observed by about 9.5 and 5.2% for the specimens F-PC5 and F-PC8, respectively, associated with RC. This lesser increment in strength is attributed to PF’s addition, which has low tensile strength and inadequate fibre bridging action. On the other hand, a higher compressive strength was noted for the specimens F-PC3, F-PC4, F-PC6, F-PC7 and F-PC9 with about 48.0, 54.4, 47.6, 20.2 and 30.5%, respectively. The best contribution of fibre combination came from the F-PC4 specimens. This phenomenon is due to a 2.6% dosage of SF at the top and bottom layers, while 1.6% at the middle layer results in higher fibre bridging action.

### 3.2. Projectile Impact

It is widely known that many structures are subjected to impact loading during bomb blasts and terrorist attacks [26]. A high-velocity impact is generated from these events and damages a structure to a large extent. During this event, many small, damaged pieces can be formed from the point of impact, affecting the surrounding structure with low-velocity. Higher compression at the top face and tensile at the specimen’s bottom face resulted in crating and scabbing. When the projectile strikes with low-velocity repeatedly on the FPAFC specimens, the projectile bounces off rather than causing local damage, which results in a small fragment being ejected out from the point of impact. The projectile pierces the specimen with more depth, apart from the crater’s spalling depth, under the repeated impacts, resulting in a penetration hole in the cylindrical shape. This penetration depth increases with the projectile repeatedly striking the specimen, leading to scabbing in its bottom face. The damaged surface area at the top and bottom surfaces was calculated through the image processing failure picture. Meanwhile, the damaged area’s diameter is measured in six directions to calculate the damaged area manually. Moreover, the thin needle is used to measure the penetration depth for every four impacts [24].

#### 3.2.1. Weight Loss

**Figure 5:**
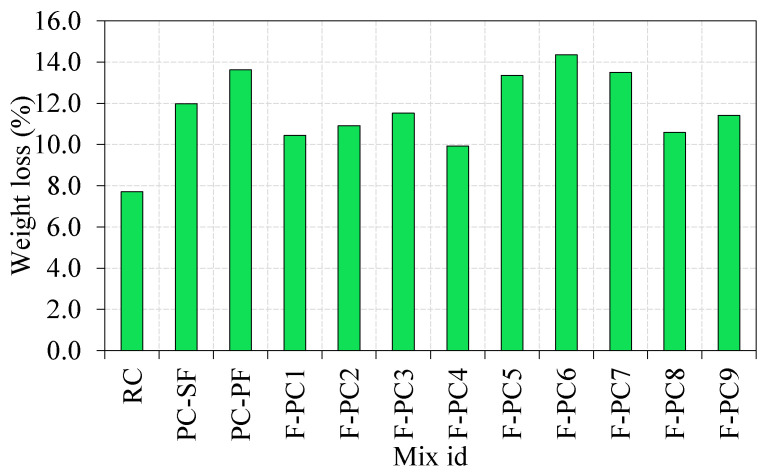
Weight loss of FPAFC specimen.

Figure 5 depicts the percentage weight loss of FPAFC specimen after failure. The specimen’s weight was measured before and after testing and the weight loss is calculated accordingly [27]. It can be noted from Figure 5 that the weight loss that happened in the RC specimen was 7.7%; this small intrinsic weight loss was due to the specimen experiencing a smaller number of impacts (12 hits). The weight losses of 12.0 and 13.6% were observed for the PC-SF and PC-PF specimens, with the corresponding numbers of impacts being 35 and 16, respectively. Likewise, a 10.4 and 10.9% weight loss were observed for the F-PC1 and F-PC2 specimens with the corresponding numbers of impacts being 20 and 22, respectively. On the other side, in the three-layered FPAFC specimens, the weight loss ranged from 9.9 to 14.4% upon failure. Regarding the number of blows of RC, the weight loss for FARPC specimens is accompanied by higher impacts, which displays an excellent projectile impact resistance. This enhancement is due to the presence of fibres that offer higher impact energy absorbance. Consequently, internal damage in the FPAFC is alleviated significantly, and thereby, weight loss upon failure was decreased, while there was an increased number of impacts.

#### 3.2.2. Penetration Depth

**Figure 6:**
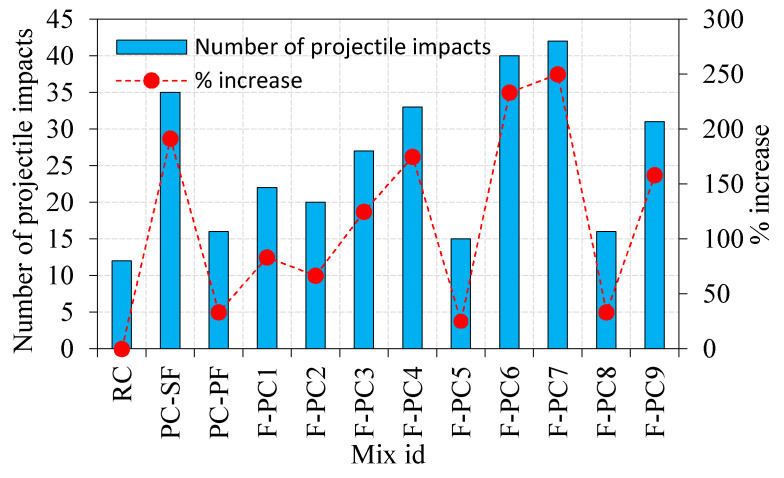
Penetration depth and the number of impacts.

The penetration depth is considered as the projectile traveled in-depth into the FPAFC specimen normal to the impact surface [24]. The penetration depth was increased with an increasing number of impacts, as illustrated in Figure 6. The projectile ejected out from the bottom specimen surface is defined as a failure in this research. The F-PC6 and F-PC7 exhibited excellent penetration resistance with the corresponding impacts being 40 and 42, respectively, as associated with RC. A total of 35 impacts was required until the failure of the PC-SF specimen. The number of blows required to eject the projectile out were 16, 22, 20, 27, 33, 15, 16 and 31 for the specimens PC-PF, F-PC1, F-PC2, F-PC3, F-PC4, F-PC5, F-PC8 and F-PC9, respectively. These values were increased by about 33, 83, 67, 125, 175, 25, 33 and 158%, compared to RC. It is worth pointing out that penetration resistance F-PC6 was much higher than FPAFC specimens. Based on previous studies [28,29], fibre addition into concrete is the more efficient strategy to enhance the tensile strength, energy absorption, hardness and shear strength. The tremendous amount of energy at the impact surface generated by a projectile was observed by fibre bridging and aggregates’ toughness [29].

#### 3.2.3. Damaged Surface Area at the Top and Bottom Face

**Figure 7:**
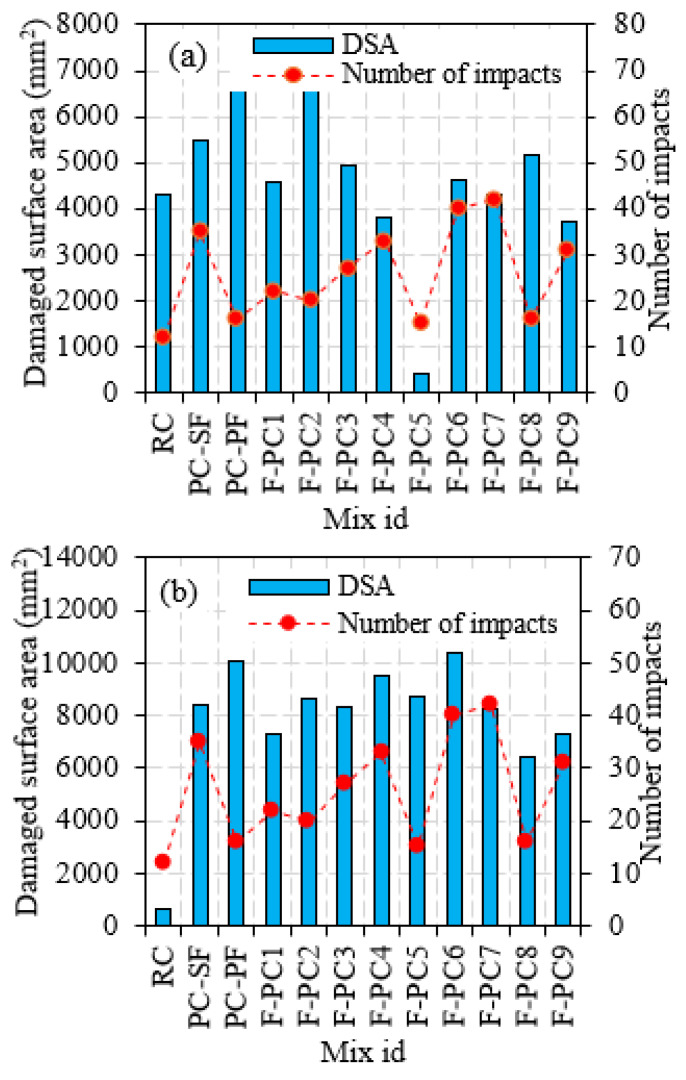
Damaged surface area (DSA): (**a**) top and (**b**) bottom.

The damaged top surface area is defined as the impacted area opening resulting from repeated projectile impacts. Fiji image J software is used for measuring the damaged area. The recorded damaged area’s images were processed through the software, and boundaries were marked with many points connected in the marking sequence. The actual surface area was calculated using the diameter of the specimen. The damaged area was measured directly from the connection points. A high-resolution camera was used to capture a specimen’s damaged surface, and images were taken clearly without any blur. This area can also be found by an equivalent diameter measured in six directions [30]. Figure 7a observed that the damaged top surface area is increased dramatically for the first ten impacts and then gradually decreased for the succeeding impacts. This is ascribed to the exceptional resistance to penetration and cracks of the FPAFC comprising SF and PF. This is the anticipated damage pattern: the formation of a small crater at the top surface during initial impacts, resulting in many small pieces being ejected due to the damage, followed by the formation of a cylindrical penetration hole [24]. The RC’s damaged area was 4293 mm^2^ and this damage happened with 12 impacts. On the other side, fibres serve as anti-penetration material in the FPARC specimens, decreasing the top surface’s damaged area [30]. For example, the PC-SF and PC-PF specimens’ damaged areas were 5463 and 6862 mm^2^, respectively. Likewise, the observed damage area for the F-PC1, F-PC2, F-PC3, F-PC4, F-PC5, F-PC16, F-PC17, F-PC8 and F-PC9 were 4592, 6667, 4940, 3842, 422, 4643, 5178 and 3719 mm^2^, respectively. There is a large difference in damage area upon failure and the observed pattern did not follow any trend. For all cases, the damaged areas are reduced by fibres, preventing specimen disintegration at the failure stage. This phenomenon is due to fibres existence near the impacts point, which allows the absorption of impact energy and lessens the degree of damage. The SF and PF hybridization effectively inhibit cracks, lessening the damaged area at the top surface. During the first impact, the sparks flew off from the point of impact, which induced high compression, and the projectile was ricocheted off by the specimen. Then, the projectile pierced into the specimens against multiple impacts.

Four damage patterns were observed in the FPAFC specimens against impact load; the crater region, region of penetration, region of crushed aggregate and region of cracking [31]. Besides, scabbing was also observed on the bottom face. Damaged bottom surface area is the key factor to describe the penetration resistant behaviour. The damaged portion area is mostly bigger in the bottom than the top surface due to the following reasons; the damaged top surface area is caused by the formation of a compressive wave by the projectile, while it occurs at the bottom surface due to tensile waves. The formation of the cracking zone can be greatly imputed to elastic stress waves [32,33]. The longitudinal compressive wave is in a sphere-shaped form and it reaches the bottom surface. The reflectivity of the tensile wave was generated. Therefore, the tensile wave amplitude exceeds dynamic tensile strength at any point of the FPAFC specimen, causing cracking and scabbing. Figure 7b depicts a damaged bottom surface area upon failure of FPAFC specimens. It is clear from Figure 7b that the RC specimen’s damaged bottom surface area was 668 mm^2^, corresponding to 12 impacts. The maximum damage area was noted in the F-PC6 specimen, followed by the PC-PF specimen, and the corresponding values were 10,373 and 10,041 mm^2^. Likewise, the damaged area for the F-PC5 and F-PC8 specimens containing only PF were 8687 and 6408 mm^2^, respectively, with the corresponding numbers of impacts being 15 and 16. The reason for this intrinsic decrement in number blows was the low tensile strength of PF and ineffective fibre bridging action. On the other side, the values for PC-SF, F-PC1, F-PC2, F-PC3, F-PC4, F-PC7 and F-PC9 were 8409, 7264, 8643, 8352, 9503, 8283 and 7331 mm^2^. In a nutshell, increasing the fibre dosage at the bottom and top layers and hybrid combination positively impacts the damaged area with an increasing number of impacts.

#### 3.2.4. Damage Ratio

**Figure 8:**
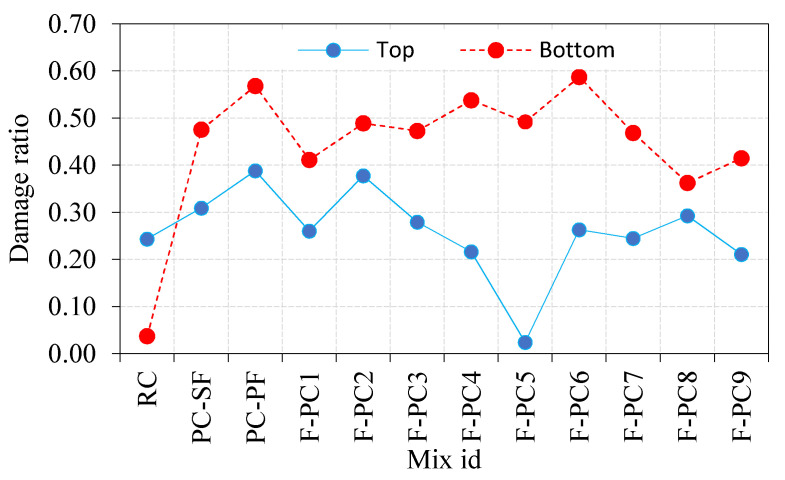
Damage ratio of FPAFC.

Figure 8 illustrates the damage ratio of the top and bottom surfaces after failure. The ratio between the damaged area of top/bottom surface after failure and the original area before applying impact load is defined as the damage ratio. It is noticeable from Figure 8 that the damaged area of the top surface is greater than the bottom surface. The observed damage ratios for the top and bottom surfaces were 0.24 and 0.04, respectively. This phenomenon was due to the projectile striking the specimen’s top surface, which created more damage, and the specimen was broken into two pieces before the spalling occurred. In contrast, the top surface damage ratio of FAPFC specimens varied from 0.02 to 0.39, while they were from 0.36 to 0.59 for the bottom surface damage ratio. The reason for these fluctuations in the damage ratio was the same as discussed in the earlier section.

#### 3.2.5. Failure Patterns

**Figure 9:**
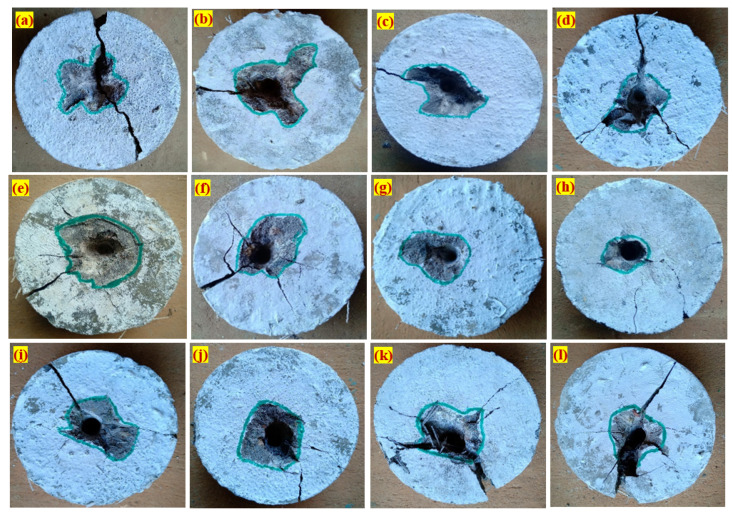
Damaged top surface area: (**a**) RC, (**b**) PC-SF, (**c**) PC-PF, (**d**) F-PC1, (**e**) F-PC2, (**f**) F-PC3, (**g**) F-PC4, (**h**) F-PC5, (**i**) F-PC6, (**j**) F-PC7, (**k**) F-PC8 and (**l**) F-PC9.

**Figure 10:**
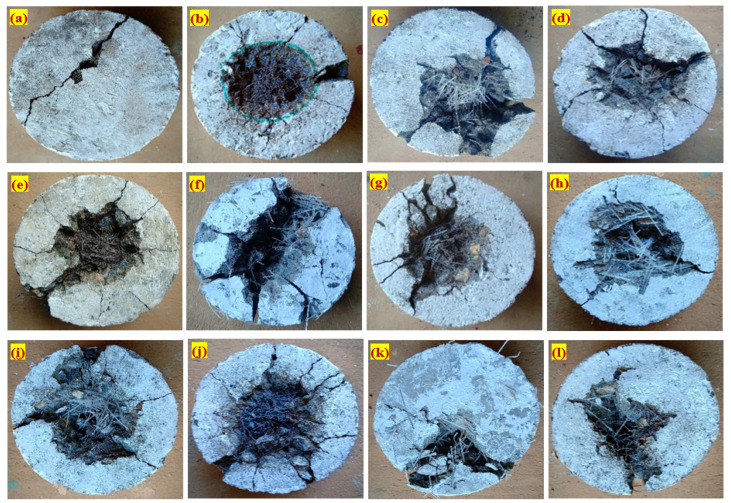
Damaged bottom surface area: (**a**) RC, (**b**) PC-SF, (**c**) PC-PF, (**d**) F-PC1, (**e**) F-PC2, (**f**) F-PC3, (**g**) F-PC4, (**h**) F-PC5, (**i**) F-PC6, (**j**) F-PC7, (**k**) F-PC8 and (**l**) F-PC9.

**Figure 11:**
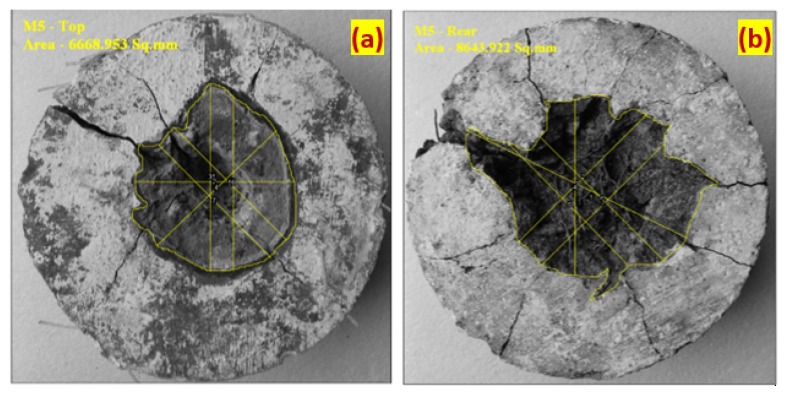
Damage area measurement through image processing (F-PC2 specimen) (**a**) Top surface (**b**) Bottom surface.

The failure pattern of the bottom and top surface against the projectile impacts is displayed in Figure 9 and Figure 10. The observed failure pattern of FPAFC is as follows; first, a small penetration joined with the damaged area happened in the top surface near the point of impact during the first few impacts. At this moment, the projectile pierced the specimen and stuck inside the specimen without bouncing back, resulting in scabbing with a punctured hole being observed. Second, an increase in the damaged area at the top surface was observed and joined with penetration depth under the number of projectiles. At this moment, penetration failure was noticed without causing any damage at the surface bottom. Third, scabbing occurred at the bottom surface and is clearly visible in the form of a puncture hole [27], with an irregular shape of the damaged area, as shown in Figure 10. At this moment, specimens were not disintegrated into many fragments; even the spalling occurred. This behaviour is attributed due to fibre bridging action, which holds the broken fragments attached to the specimen. Elastic wave propagation in compression and tensile nature are the main reason for cracking the specimen, resulting in crating and scabbing occurring on the specimen [24]. From a practical standpoint, an exceptional impact resistance capacity is achieved from FPAFC and can be considered a good fit. All specimens’ failure patterns were captured with a high-resolution camera, and the image process was employed to find the damaged area at the top and bottom surface. The image processed picture of the damaged top and bottom surface areas are illustrated in Figure 11.

## 4. Evaluation of the Ejected Mass from the Top Surface of FPAFC

**Figure 12:**
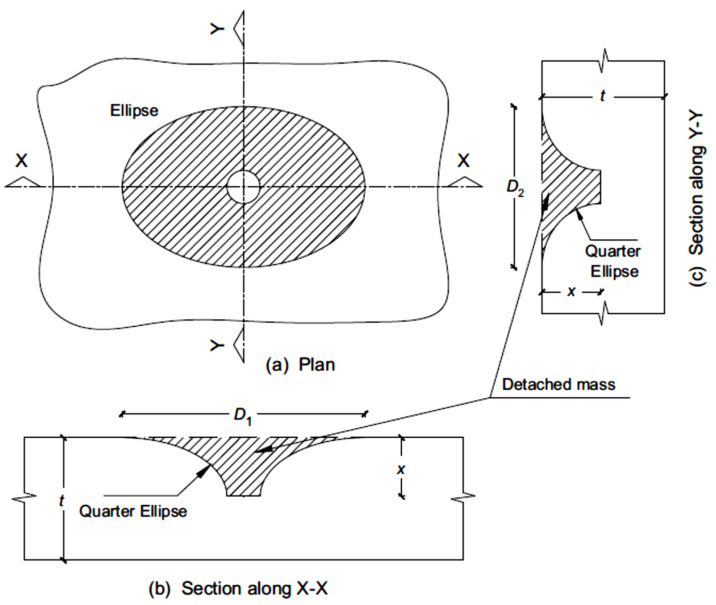
Idealisation of ejected composite mass from the front surface of FPAFC (x = depth of penetration; t = target thickness of; D1 and D2 are diameters measured in different directions of the destroyed area at the front surface, respectively) [34]. (**a**) Plan (**b**) Section along X-X (**c**) Section along Y-Y

**Figure 13:**
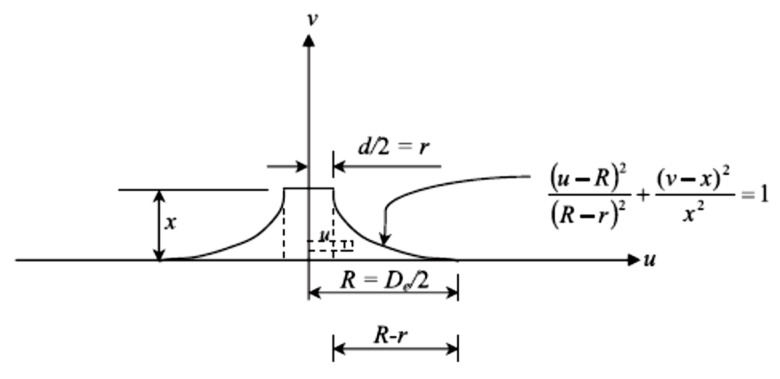
Idealized illustration for the assessment of ejected composite mass from the top surface of FPAFC [34].

The evaluation of ejected mass from the specimen’s top surface was predicted in this study using a simplified analytical model. The prediction of the top surface fragmented mass, with the assumption of the mass of the cylindrical tunnel created during the perforation, and the mass of an elliptical cylinder were equal. This elliptical cylinder diameter is assumed to be the same as the equivalent average diameter measured in six directions. Table 3 demonstrates the average diameter of the top damaged portion measured in six directions. The diameter was measured using a digital vernier calliper. The ejected mass from the top surface of FPAFC specimens’ targets was evaluated by idealizing the joint damage of the damaged area and punctured hole, as shown in Figure 12 and Figure 13.

It was assumed that the top surface damaged area has an elliptical shape and the damaged shape area at the penetration depth is circular. It was also assumed that the projectile diameter is the same as the punctured hole diameter. The additional assumption was made that the difference in shape from the top surface damaged diameter to penetration depth is considered elliptic. To simplify the difficulties, the ellipse shape at the top surface was replaced by an equivalent diameter. The ejected mass from the top surface can be calculated using Equation (1) as follows [34],

(1) $$M=\gamma \left[\int_{v=0}^{x}\int_{u=r}^{u}2\pi \;u\;dudy+\frac{\pi }{4}d^{2}x\right]$$

From the Equation (1) first term is realigned by integration and yields the following equation [34],

(2) $$M=\frac{\pi }{4}\gamma \left[\;xd^{2}\int_{v=0}^{x}\left(4u^{2}-d^{2}\right)dv\right]$$

With this assumption of the damaged top surface area around the hole as a quadrant of an ellipse, the variable *u* can be determined. The line of the damaged area at the top surface can be attained from Equation (3) as follows,

(3) $$\frac{{\left(u-R\right)}^{2}}{{\left(R-r\right)}^{2}}+\frac{{\left(v-x\right)}^{2}}{x^{2}}=1$$

Equation (4) yields further,

(4) $$u=\frac{D_{e}}{2}+\frac{\left(D_{e}-d\right)}{2x}\sqrt{2xv-v^{2}}$$

Equation (4), integrated with the substitution of the expression *u*, yields,

(5) $$M=\gamma \frac{\pi x}{24}\left[4\lambda d^{2}+D_{e}\left\{2\left(5D_{e}-4d\right)-3\pi \left(D_{e}-d\right)\right\}\right]$$

where *γ* is the FPAFC density, *D_e_* and *R* are the diameter and radius of the damaged surface area, respectively, *d* and *r* are the diameter and radius of the punctured hole, which is considered equal to the projectile radius and diameter, *x* is the penetration depth, u and *v* are the defined variables shown in Figure 13. A newly added parameter λ has been displayed in Equation (5) and it is considered as zero because the FPAFC target gets perforated; otherwise, its value is unity. This analytical model contains a parameter that was experimentally measured after the test (diameter *D_e_*, depth *x*) to approximately predict the ejected mass. This eradicated the difficulties of measuring the ejected mass from the top surface after the failure, accompanied by punctured holes and bottom-damaged areas in the specimen.

**Figure 14:**
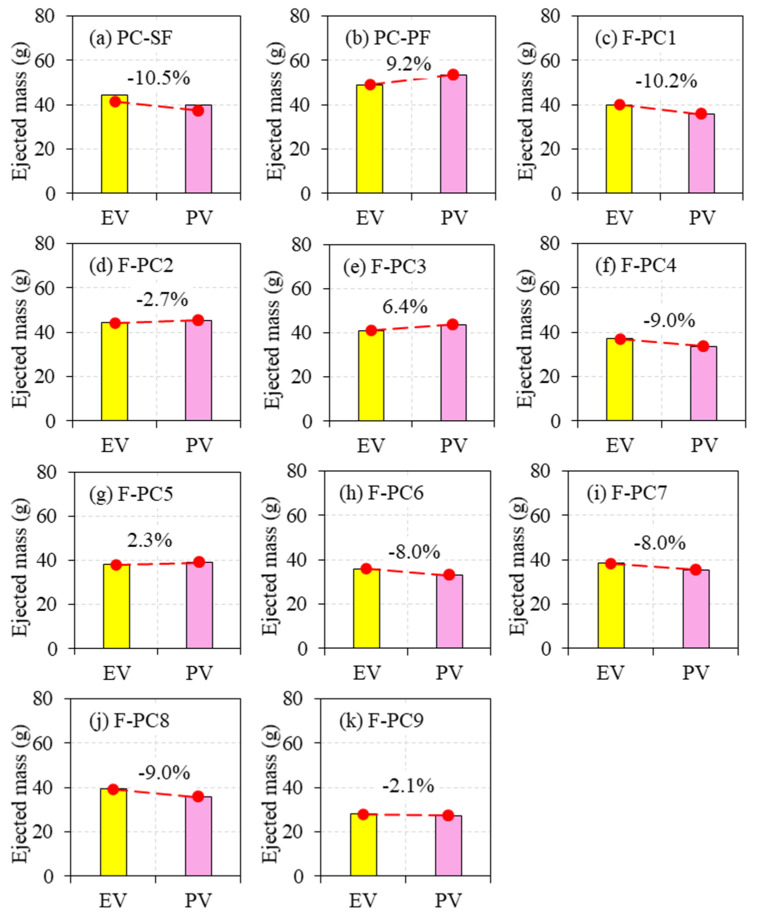
Comparison of ejected mass of experiment value (EV) and predicted value (PV). (**a**) PC-SF, (**b**) PC-PF, (**c**) F-PC1, (**d**) F-PC2, (**e**) F-PC3, (**f**) F-PC4, (**g**) F-PC5, (**h**) F-PC6, (**i**) F-PC7, (**j**) F-PC8 and (**k**) F-PC9.

The comparison was made between the ejected mass observed from the experimental test and computed through the analytical model. The difference in results in terms of percentage between the experimental value and predicted value for all tested specimens are exemplified in Figure 14a–k. It is noticeable from Figure 14 that, in many cases, the predicted values were slightly less than the experimental values. The highest error in terms of percentage between the experimental and predicted values was 10.5, which shows the accuracy of the analytical model. This small error occurred due to the idealization uncertainties, small mass involvement, humanmade errors during ejected mass measurements and fall height. It is hand-actuated work and is, therefore, challenging to control. In a nutshell, adding fibres in concrete can significantly reduce the damage level of the concrete [35,36,37,38,39].

## 5. Conclusions

The key findings are drawn up, on the basis of the above discussion, as follows:

1. The SF had a positive contribution in enhancing compressive strength compared to PF. The maximum compressive strength noted from the PC-SF specimen increased by about 59.5% compared to the RC specimen. SF’s addition in a single layer exhibited higher compressive strength than two/three-layered FPAFC. This is ascribed to effective bridging action resulting in microcrack development before failure, thus enhancing crack resistance and propagation.
2. The SF and PF hybridization in three-layered FPAFC exhibited a superior compressive strength. For example, SF and PF’s hybridization in F-PC3 and F-PC6 specimens tend to rise in compressive strength by 48.0 and 47.6%, respectively. The reason for this intrinsic increment is SF and PF jointly bridging the micro-cracks and postponing their growth as a result of good fibre and matrix bonding.
3. The increasing number of impacts mostly accompanied the weight loss of FPAFC specimens. The weight loss of FPAFC specimens against projectile impacts ranged from 9.1 to 14.4%, with the corresponding number of impacts ranging from 33 to 40. The number of blows required to eject the projectile was assessed. The specimens F-PC6 and F-PC7 exhibited excellent penetration resistance with the corresponding impacts of 40 and 42, respectively, as associated with RC.
4. The damaged top surface area ranged from 422 to 6862 mm^2^ for the FPAFC specimens. The damaged areas are reduced by fibres, which prevent the specimens disintegration upon failure. This phenomenon is due to fibres existence near the impact point, which allows the absorption of impact energy and lessens the degree of damage. The maximum damaged bottom surface area was noted in the F-PC6 specimen, and the corresponding value was 10,373 mm^2^. Increasing the fibre dosage at the top and bottom layers of FPAFC and hybrid combination alleviates the damaged bottom surface area with an increasing number of impacts.
5. Two different failure patterns were observed. The RC specimen was broken into two pieces under projectile impacts, which shows its brittle nature. Conversely, all FPAFC specimens had crating and scabbing at the top and bottom surfaces. This is due to the elastic wave propagation, in which compression and tensile nature are the main reasons for cracking of the specimen, resulting in crating and scabbing occurring on the specimen surfaces.
6. A simple analytical model was used to evaluate the theoretical ejected mass from the top surface of the FPAFC specimens. The analytical model’s predicted values were in good agreement with the experimental values, with the maximum percentage error being 10.5%. Therefore, the used model for finding ejected mass is practicable and rational when used for concrete composites.

## Figures and Tables

**Table 1:** Mix composition of FPAFC.

Mix Id	s/c	w/c	Fiber Dosage (%)						SP
			I Layer		II Layer		III Layer		(%)
			SF	PF	SF	PF	SF	PF	
RC	1	0.45	0						0.3
PC-SF			2.4 SF						0.4
PC-PF			2.4 PF						0.4
F-PC1			2.4 SF			2.4 PF			0.4
F-PC2			2.4 PF			2.4 SF			0.4
F-PC3			1.2	1.2	1.2	1.2	1.2	1.2	0.4
F-PC4			2.8	0	1.6	0	2.8	0	0.4
F-PC5			0	2.8	0	1.6	0	2.8	0.4
F-PC6			1.4	1.4	0.8	0.8	1.4	1.4	0.4
F-PC7			3.6	0	0	0	3.6	0	0.4
F-PC8			0	3.6	0	0	0	3.6	0.4
F-PC9			1.8	1.8	0	0	1.8	1.8	0.4

**Table 2:** Compressive strength of FPAFC.

	Compressive Strength MPa			
Mix ID	Specimen 1	Specimen 2	Specimen 3	Average
RC	32.18	29.09	33.85	31.71
PC-SF	48.9	52.3	50.5	50.57
PC-PF	34.19	41.18	37.51	37.63
F-PC1	39.49	41.84	38.64	39.99
F-PC2	39.92	35.74	41.38	39.01
F-PC3	44.15	46.92	49.75	46.94
F-PC4	48.05	49.58	49.27	48.97
F-PC5	32.52	36.88	34.74	34.71
F-PC6	45.71	45.79	48.92	46.81
F-PC7	34.81	38.23	41.32	38.12
F-PC8	34.44	32.26	33.41	33.37
F-PC9	42.71	38.32	43.13	41.39

**Table 3:** The average diameter of damaged portion measured in six directions.

Mix ID	PC-SF	PC-PF	F-PC1	F-PC2	F-PC3	F-PC4	F-PC5	F-PC6	F-PC7	F-PC8	F-PC9
De (mm)	57.8	59.8	47.5	56.5	56.8	47.6	28.7	50.1	54.1	45.9	39.6

## Data Availability

Data sharing not applicable.
